# The Role of Serum Anti-Mullerian Hormone Measurement in the Diagnosis of Polycystic Ovary Syndrome

**DOI:** 10.3390/diagnostics13050907

**Published:** 2023-02-27

**Authors:** Mala S. Sivanandy, Sierra K. Ha

**Affiliations:** 1 PCOS Center, Division of Endocrinology, Beth Israel Deaconess Medical Center, Boston, MA 02215, USA; 2 Harvard Medical School, Boston, MA 02115, USA

**Keywords:** polycystic ovary syndrome, anti-mullerian hormone, diagnosis, hyperandrogenism, oligomenorrhea

## Abstract

Polycystic ovary syndrome (PCOS) is a common endocrinological disorder in women with significant reproductive, metabolic, and psychological health implications. The lack of a specific diagnostic test poses challenges in making the diagnosis of PCOS, resulting in underdiagnosis and undertreatment. Anti-Mullerian hormone (AMH) synthesized by the pre-antral and small antral ovarian follicles appears to play an important role in the pathophysiology of PCOS, and serum AMH levels are often elevated in women with PCOS. The aim of this review is to inform the possibility of utilizing anti-Mullerian hormone either as a diagnostic test for PCOS or as an alternative diagnostic criterion in place of polycystic ovarian morphology, hyperandrogenism, and oligo-anovulation. Increased levels of serum AMH correlate highly with PCOS, polycystic ovarian morphology, hyperandrogenism, and oligo/amenorrhea. Additionally, serum AMH has high diagnostic accuracy as an isolated marker for PCOS or as a replacement for polycystic ovarian morphology.

## 1. Introduction

Polycystic ovary syndrome is the most common endocrine disorder in women of reproductive age, with an estimated prevalence of 8–13% [1]. It is also the most common cause of anovulatory infertility [2]. It is a heterogeneous disorder with multiple different phenotypes and affects both adolescent and adult women. The syndrome was first described by two gynecologists, Irving Stein and Michael Leventhal, in 1935 [3], and there is no single diagnostic test available for PCOS to date. The syndrome encompasses gynecological symptoms such as oligomenorrhea, amenorrhea, infertility; dermatological symptoms such as hirsutism, acne, alopecia; and metabolic complications such as prediabetes, type 2 diabetes [4], obesity, dyslipidemia [5], non-alcoholic fatty liver disease (NAFLD) [6], metabolic syndrome [7], and obstructive sleep apnea [8]. There is a high prevalence of anxiety and depression in women with PCOS [9]. Women with PCOS are also at higher risk of developing endometrial hyperplasia and endometrial cancer [10,11]. The term polycystic ovary syndrome is considered as a misnomer as there are no epithelial cysts in the ovaries and the polycystic appearance comes from the antral follicles [12]. Not only is the term confusing, the pathophysiology of polycystic ovary syndrome is also complex and involves reproductive and metabolic dysfunction [13]. Aberrant GnRH pulsatility [14], abnormal gonadotropin secretion [15], and ovulatory dysfunction are some of the factors involved in the pathogenesis of PCOS in addition to hyperandrogenism and insulin resistance. Low-grade chronic inflammation with high levels of C reactive protein (CRP), endothelial dysfunction [16], and high oxidative stress [17] contribute further to the complexity of pathogenesis of polycystic ovary syndrome.

## 2. Diagnostic Criteria for PCOS

**Figure 1:**
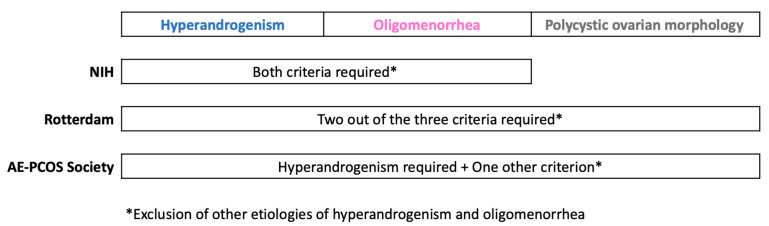
Current diagnostic criteria for PCOS.

Since the pathogenesis is not fully understood, the diagnosis of PCOS is also neither simple nor straightforward, leading to the development of several sets of diagnostic criteria (Figure 1). Although PCOS can present with multiple different symptomatology, at a National Institutes of Health (NIH)-sponsored conference on PCOS in 1990, attendees voted highly for hyperandrogenism and chronic anovulation as potential diagnostic features for PCOS [18]. Thus, the NIH criteria proposed in 1990 require the presence of oligomenorrhea and hyperandrogenism to make a diagnosis of PCOS while excluding other causes of hyperandrogenism and oligomenorrhea [19]. The common differentials are hyperprolactinemia, thyroid dysfunction, late-onset non-classic congenital adrenal hyperplasia, Cushing’s syndrome, primary ovarian insufficiency, hypothalamic amenorrhea, pregnancy, etc. In 2003, at the Rotterdam consensus meeting, polycystic ovarian morphology on ultrasound was added to the above criteria considering the heterogeneous nature of PCOS and to reduce delay in diagnosis, which in turn can lead to delayed treatment [20]. In 2006, the Androgen Excess and PCOS Society defined PCOS as a disorder of hyperandrogenism and oligomenorrhea (or) polycystic ovarian morphology (or) both [21]. Exclusion of other conditions that mimic PCOS involves performing specific diagnostic tests for each of the differentials as appropriate.

## 3. Challenges with the Current Diagnostic Criteria

Primarily, having different sets of criteria is a source of confusion, not only for clinicians but also for patients. In addition, there are multiple challenges with each of the three diagnostic elements of PCOS: hyperandrogenism, oligomenorrhea, and polycystic ovarian morphology on ultrasound. Clinically, hyperandrogenism can manifest as hirsutism, acne, or alopecia. Acne cannot be considered as a diagnostic criterion in adolescents unless it is severe. While modified Ferriman–Gallwey (mFG) and Ludwig visual scoring systems are used to assess the severity of hirsutism and alopecia, respectively, no such universal visual scoring system is available to assess the severity of acne. There is also marked ethnic variation in the manifestation of hirsutism, requiring consideration of different cut-offs for different ethnicities [22], and women self-treating hirsutism can further complicate the clinical assessment. Biochemically, there are issues with the laboratory assays available for measuring free and total testosterone concentrations. Nearly 99% of serum testosterone is bound to binding proteins such as sex hormone-binding globulin (SHGB) and albumin, and only 1% circulates in the unbound free form, which is the active form of testosterone. Though free testosterone is best measured by equilibrium dialysis, it is not widely available in many laboratories because of its high cost and the labor-intensive process involved. The commonly available immunoassays that directly measure free testosterone levels are less accurate. Sex hormone-binding globulin (SHBG) levels can be low in women with PCOS which can affect the measurement of serum total testosterone and calculation of free androgen index (FAI) and bioavailable testosterone [23]. In some women with polycystic ovary syndrome, androgens other than testosterone such as DHEAS (dehydroepiandrosterone sulfate) or androstenedione can be the only hormone elevated.

Oligomenorrhea is physiological around menarche [24] and menopause, and a history of regular menstrual cycles does not always exclude oligo-ovulation. The definition of oligomenorrhea varies depending upon where the woman is in the reproductive spectrum. One to three years post menarche, adolescents with menstrual cycles < 21 or >45 days (or) >90 days for any one cycle are considered oligomenorrheic. In women three years post menarche up to menopause, adults with menstrual cycles < 21 or >35 days (or) less than eight cycles per year are considered oligomenorrheic [25]. The polycystic ovarian morphology criterion cannot be used in women with gynecological age of less than eight years nor in perimenopausal women. To detect ≥20 follicles per ovary in order to fulfill the criterion, a higher frequency 8 MHz transducer is required [25,26]. Though transvaginal ultrasound accurately measures ovarian volume and antral follicle count, it is not an appropriate procedure for women who have never been sexually active. Transabdominal ultrasound poses difficulty in accurately detecting antral follicles, especially in women with obesity. So, clinicians continue to face multiple challenges in making the diagnosis of PCOS, and there has been a constant search for a better or alternative diagnostic test or diagnostic criterion.

## 4. Anti-Mullerian Hormone

AMH is a unique dimeric glycoprotein that was first described by Alfred Jost in the 1940s [27]. It belongs to the TGFβ super family [28,29] and plays an important role in sexual differentiation [30] and regulation of folliculogenesis [31]. It derives its name because of its ability to inhibit the development of mullerian duct structures in the male fetuses [30]. AMH is composed of two identical glycoprotein subunits, each of which has a larger N-terminal prodomain and a smaller C-terminal mature signaling domain, both connected by disulfide bridges. Pre-proAMH is a precursor molecule that undergoes proteolytic cleavage, producing biologically inactive proAMH which then yields the biologically active form of AMH [32]. AMH attaches to specific receptors on cells of target tissues. The C-terminal of AMH binds to the extracellular domain of AMH type 1 and type 2 serine/threonine kinase receptors, producing an intracellular Smad signal, which in turn regulates target gene transcription [33,34].

**Figure 2:**
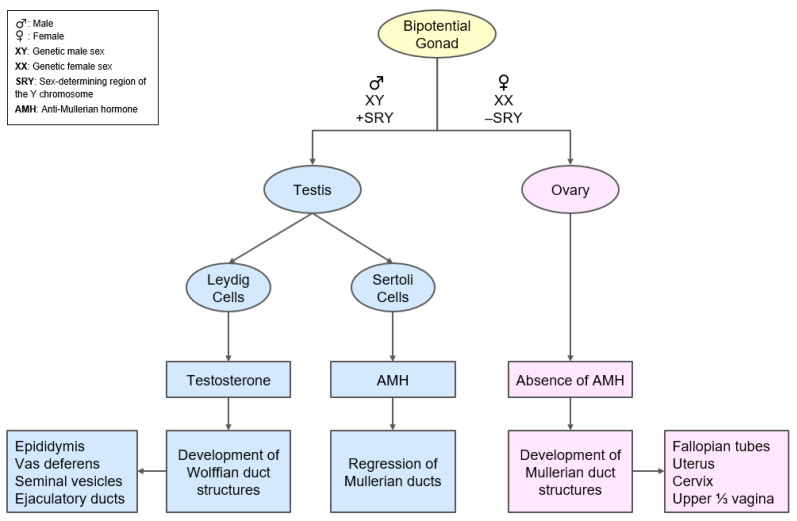
Schematic representation of the role of AMH in the sexual differentiation of the fetus.

Anti-Mullerian hormone plays a major role in sex differentiation (Figure 2). The gonads are indifferent until the sixth week of fetal life. Genetic sex is determined by the sex chromosomes. The sex determining region of the Y chromosome (SRY) in male (XY) fetus allows the indifferent gonad to develop into testes. The Leydig cells secrete testosterone that stimulates development of Wolffian duct structures and Sertoli cells secrete Anti-mullerian hormone that suppresses the development of Mullerian duct structures. In female (XX) fetuses, the absence of SRY allows the gonads to develop into ovaries, and the absence of AMH in early fetal life allows Mullerian ducts to develop into fallopian tubes, uterus, cervix, and the upper third of the vagina [35].

The gene for AMH is on the short arm of chromosome 19 [36], and the gene for the AMH receptor type 2 is on the long arm of chromosome 12 [37]. AMH expression is observed in granulosa cells of primary, secondary, and small (<4 mm diameter) antral follicles while it is absent in primordial and larger (>8 mm diameter) antral follicles [38]. Primordial follicles start appearing in utero around 15 weeks of gestation in female fetuses, and during growth and development, most follicles become atretic through a hormonally regulated ligand-receptor system and associated granulosa cell apoptosis [39]. The remaining stay dormant until actively recruited into the growing pool for maturation and ovulation [40]. Primary follicles have a single layer of cuboidal granulosa cells surrounding the oocyte, and early secondary follicles acquire a second layer. The antral follicles (also called tertiary follicles) derive their name from having a fluid-filled space called the antrum and have multiple layers of granulosa cells [41].

Follicle-stimulating hormone (FSH) [42] and intraovarian regulators such as kit-ligand (SCF stem cell factor) [43] and neurotrophins [44] play an important role in initial primordial follicle recruitment. Anti-Mullerian hormone regulates ovarian folliculogenesis by inhibiting the recruitment of primordial follicles from the pool. It is considered as a marker of ovarian reserve. AMH levels decrease with chronological age and after menopause [45,46]. AMH elimination from the body follows first order pharmacokinetics and reaches approximately 90% after four days and 95% after approximately five days, and the level becomes undetectable eight days after salpingo-oophorectomy in premenopausal women. The mean terminal half-life of AMH is 27.6 ± 0.8 h with a range from 12.3 to 39.9 h in single cases [47].

## 5. Anti-Mullerian Hormone and PCOS

In women with PCOS, there is increased pulsatile frequency of GnRH (gonadotropin-releasing hormone) that stimulates LH (luteinizing hormone), which in turn increases ovarian theca cell production of androgens [18]. Additionally, there is augmented androgen production due to increased activity of multiple steroidogenic enzymes in theca cells [48]. Relative FSH deficiency impairs aromatization of androgens [18].

Women with PCOS are noted to have higher levels of Anti-Mullerian hormone [49]. Serum AMH levels are significantly higher in normogonadotropic anovulatory women (World Health Organization class 2, or WHO2), especially those with polycystic ovarian morphology compared to age-matched normoovulatory premenopausal women [50]. Mean AMH levels in in vitro ovarian granulosa cells from anovulatory PCOS women are 75-fold higher compared to in vitro ovarian granulosa cells from age matched normal ovulatory controls [51]. In cells from PCOS women, luteinizing hormone (LH) increased AMH and follicle-stimulating hormone (FSH) decreased AMH [51].

In PCOS women, mean follicular fluid AMH levels are 60-fold higher than their serum AMH levels [52]. Follicular fluid aspirated from 3–4 size matched 4–8 mm follicles from women with anovulatory PCOS has significantly higher AMH levels compared to age matched normally ovulating women, raising the possibility of increased AMH production per follicle [52]. AMH levels are reported to correlate with testosterone, free androgen index, LH, mean ovarian volume, and follicle number on transvaginal ultrasound in WHO2 patients [50]. Higher expression of AMH and AMH receptor 2 is noted in granulosa cells from PCOS women [53]. In human granulosa lutein cells, AMH inhibits FSH-induced adenylyl cyclase activation, expression of aromatase, and production of estradiol, and it reduces messenger RNA expression of FSH receptors [54,55]. However, there is no effect noted on basal aromatase expression [55,56]. AMH receptor 2 is also expressed in approximately more than half of hypothalamic GnRH neurons in adults and during embryonic development. Increased GnRH secretion and resultant increased LH secretion are noted in mice in response to exogenous AMH, suggesting a role for AMH in regulation of GnRH neuron excitability and secretion [57]. In animal studies, AMH 2 receptor expression is seen in endothelial cells and specialized hypothalamic glial cells called tanycytes that are known to regulate GnRH secretion [58,59].

**Figure 3:**
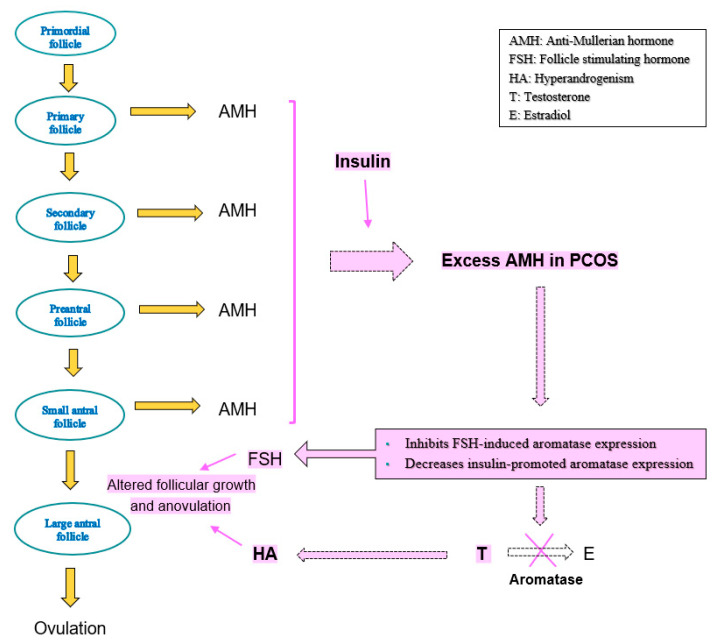
Schematic representation of ovarian follicular development, AMH secretion, excess production in PCOS, its possible inhibitory effect on FSH and aromatase, and contribution to hyperandrogenism and altered follicular growth.

A high concentration of AMH in anovulatory PCOS could be the cause of an exaggerated inhibitory effect on follicular growth [54]. AMH also inhibits gonadotropin-stimulated CYP19A and P450scc gene expression in cultures of human granulosa lutein cells, suggesting that it may have a regulatory role in ovarian steroidogenesis as well [56]. One hypothesis proposed is that hyperandrogenism in PCOS hypersensitizes granulosa cells to FSH, resulting in excessive preantral follicular growth and AMH expression, which in turn inhibits FSH-induced aromatase expression (Figure 3). This results in altered antral follicular growth causing anovulation [60]. Increased AMH expression is also noted in response to insulin in luteinized granulosa cells from PCOS women. However, adding AMH decreases insulin-promoted aromatase expression [53]. All of the above imply that the high local concentration of AMH in addition to hyperandrogenism due to intrinsic theca cell dysfunction [48,61] could alter the follicular microenvironment and play an important role in the pathogenesis of PCOS [60].

## 6. Anti-Mullerian Hormone Levels and Assays

AMH is secreted by the testicular Sertoli cells in early fetal life in male fetuses whereas the ovarian granulosa cells in female fetuses start secreting AMH around the 36th week of gestation [62]. Throughout the life cycle, AMH is typically elevated in males compared to females. AMH level in the first month of neonatal life is significantly higher in boys (median 57.2 ng/mL; 5th–95th percentile 23.8–124) than girls (median < 0.4 ng/mL; 5th–95th percentile < 0.4–1.3), and AMH continues to increase during the first year of life. [63]. Maximum AMH is attained at age 15.8 in girls, and plateaus until age 25, then declines with age and becomes undetectable after menopause [46,64]. Based on a systematic review and meta-analysis of 11 studies, serum AMH level is higher in the follicular phase compared to the luteal phase in women with regular menstrual cycles [65]. AMH levels decrease during the course of pregnancy (approximate median values 1.69, 0.8, and 0.5 ng/mL during the first, second, and third trimesters, respectively) and after delivery, then levels increase over the next four postpartum days [66]. In reproductive age group women with PCOS and morbid obesity (BMI > 40 kg/m^2^), serum AMH level is significantly higher compared to weight-matched controls without PCOS [67]. However, in another study, obese PCOS women have significantly lower AMH levels compared to lean PCOS women, in each of the A, B, and C phenotypes (A—hyperandrogenism, oligo-anovulation, and polycystic ovarian morphology; B—hyperandrogenism and oligo-anovulation; C hyperandrogenism and polycystic ovarian morphology) of PCOS [68].

AMH can be measured in the serum as well as the follicular fluid, and immunoassays, both manual and automated, have been available for more than two decades. In women with regular ovulatory menstrual cycles, there are multiple forms of variability for the same AMH assay, including inter-participant, inter-cycle, and intra-cycle variability [69]. When three different AMH assays (Gen II-Beckman Coulter, picoAMH-Ansh Labs, and Elecsys-Roche) were compared, the inter-assay correlation in women with PCOS was stronger in the low (<2.8 ng/mL) and high (>7.04 ng/mL) range serum AMH level subgroups [70]. The variability in antibody specificity, the presence of different biologically active AMH isoforms, the analytical interference of some assays by complements, and the unavailability of an international standard to calibrate are still some of the laboratory issues associated with AMH measurement [71]. In 2021, though the commutability data did not support 16/190 (with a content of 489 ng/ampoule) to be an international reference standard, it has been accepted as a WHO reference reagent for human recombinant AMH [72]. Since AMH levels are approximately 23% lower in women using oral hormonal contraceptive compared to non-users [73], a washout period of 2–3 months off OCPs (oral contraceptive pills) may be required to accurately assess AMH levels. Additionally, pretreatment with medications such as Metformin can decrease AMH levels in women with PCOS [74]. 

## 7. AMH as an Alternative to Polycystic Ovarian Morphology (PCOM)

AMH has been suggested as an alternative for polycystic ovarian morphology (PCOM) in the diagnosis of PCOS, given that there are higher levels of AMH in women with PCOM compared to those with normal ovaries [75,76,77,78]. The two components of polycystic ovarian morphology are antral follicle count (also frequently referred to as follicle number per ovary) and ovarian volume. Serum AMH correlates specifically with antral follicle count and follicle number per ovary in the context of PCOS [75]. This predictive value of AMH for the components of PCOM can be applied to the current criteria for diagnosing PCOS. We examined the studies reviewed by Anand et al. in 2022 for those that tested serum AMH as a replacement for PCOM in the Rotterdam criteria. Thus, a diagnosis of PCOS was based on having two of three features of either oligo/amenorrhea, hyperandrogenism, or AMH above a cut-off threshold. These studies demonstrated that replacement of PCOM in the Rotterdam criteria by serum AMH level can accurately predict the presence of PCOS, with area under the ROC curve (AUC) ranging from 0.927–0.994, sensitivities of 78–100%, and specificities of 88–100% (Table 1) [78,79,80,81,82,83]. While these studies examined the predictive value of serum AMH using different cut-offs, these data suggest a possible correlation of serum levels of AMH with PCOM and PCOS. Anti-Mullerian hormone appears to be more sensitive in women with classic anovulatory PCOS compared to the ovulatory and non-hyperandrogenic phenotypes [84]. Since AMH is secreted by granulosa cells of pre-antral and small antral ovarian follicles, patients with PCOM may have higher serum AMH levels. Thus, serum AMH has a potential value as an alternative for the detection of PCOM in clinical practice to diagnose PCOS.

## 8. AMH as an Alternative to Hyperandrogenism

Given its utility as a marker of PCOS, serum AMH may be considered as an alternative to hyperandrogenism. Various factors lead to hyperandrogenism in PCOS, which in turn causes higher serum AMH. Higher levels of serum androgens, specifically total testosterone, are associated with increased production of AMH in PCOS patients [49,85,86,87,88,89]. The average AMH in women with PCOS with all three features of Rotterdam criteria has been shown to be higher than those with only features of oligo/amenorrhea and PCOM (without hyperandrogenism), suggesting that serum AMH correlates strongly with hyperandrogenism [90]. However, others have found that a higher cut-off of AMH was necessary when used as a substitute for hyperandrogenism in criteria for PCOS. In a study of 211 Caucasian women, a threshold of 45 pmol/L (6.3 ng/mL) but not 29 pmol/L (4.1 ng/mL) for AMH substituted for hyperandrogenism resulted in effective diagnosis of PCOS [75]. While AMH could be evaluated as a potential replacement for hyperandrogenism in PCOS criteria, the benefit of this is unclear given a higher threshold value for AMH is necessary to allow for diagnosis. Additionally, the relationship between AMH and testosterone and other biochemical markers of hyperandrogenism remains unclear based on current evidence [85,86,87,88,89,90,91,92]. No studies or analyses to date have specifically investigated the diagnostic accuracy of AMH in replacing hyperandrogenism for PCOS.

## 9. AMH as an Alternative to Oligo/Amenorrhea (OA)

Serum AMH may also be a marker of ovulatory dysfunction, including both oligomenorrhea and amenorrhea. Fewer studies have explored the possibility of using AMH as an alternative to oligo/amenorrhea criterion in PCOS. There are some reports of increased serum AMH levels associated with oligo/amenorrhea [75,79,93]. One study of 148 women demonstrated that substituting AMH with a cut-off of 3.19 ng/mL for oligo/amenorrhea in the Rotterdam criteria could accurately diagnose PCOS, with an AUC 0.938, sensitivity 81%, and specificity 100% [79]. Additionally, AMH has the advantage of being able to distinguish oligo/amenorrhea caused by PCOS versus premature ovarian failure or hypergonadotropic hypogonadism [94]. For younger women near the age of menarche, polycystic ovarian morphology cannot be assessed for 8 years and hyperandrogenism is difficult to distinguish given the extensive presence of acne in pubescent females [91]. Thus, given the difficulties of diagnosing PCOS with other criteria, a reliable predictor of oligo/amenorrhea in the form of serum AMH could be helpful diagnostically in this age group.

## 10. AMH as a Predictor of PCOS

The pathogenesis of PCOS is closely linked with AMH, raising the question of whether AMH can be used on its own as a predictor of PCOS. Prior studies have found AMH was not suitable as a screening tool for PCOS independent of other diagnostic criteria, as it may lead to inaccurate diagnoses for women who only have two features of PCOS or women without PCOS who have one of the features of the Rotterdam criteria [80]. However, in a recent review and meta-analysis of the diagnostic accuracy of serum AMH in PCOS including 41 studies with a total of 13,509 subjects, Anand et al. found that AMH on its own could predict PCOS with a sensitivity of 78%, specificity of 87%, and area under the ROC curve (AUC) of 0.89 [95]. Further subgroup analysis revealed a cut-off value of 4.8 ng/mL was as accurate as a higher cut-off value for PCOS detection. Overall, this suggests that AMH has potential as a reliable and effective diagnostic test for PCOS. Additionally, further support for the utility of serum AMH comes from studies showing the rate of age-related decline in AMH being lower in PCOS compared to non-PCOS women [96], indicating that it may be a valuable marker across multiple age groups.

## 11. Conclusions

Since the diagnosis of PCOS is not straightforward and based on several sets of criteria and the exclusion of other differentials, there is an ongoing search for a reliable diagnostic test for PCOS. Anti-Mullerian hormone secreted by the ovarian pre-antral and small antral follicles seems to have an important role in the pathophysiology of PCOS, and there is a correlation between serum AMH level and antral follicle count on ultrasound. In this review, we discussed the studies that investigated the sensitivity and specificity of AMH both as a predictor for PCOS and as an alternative to each of Rotterdam’s diagnostic criteria: polycystic ovarian morphology on ultrasound, oligo/amenorrhea, and hyperandrogenism. Overall, serum AMH alone or as a replacement for PCOM may have a high sensitivity and specificity to diagnose PCOS. However, there remains limited support for AMH as a replacement for oligo/amenorrhea or hyperandrogenism. Assessing the feasibility of replacing AMH for oligo/amenorrhea or hyperandrogenism with further studies, addressing the current laboratory and other challenges of AMH measurement, and further clarifying the pathophysiology of PCOS may allow serum AMH to be a useful diagnostic test for women with polycystic ovary syndrome.

## Figures and Tables

**Table 1:** Predictive value of AMH alone or AMH replacing polycystic ovarian morphology (PCOM) or oligo/amenorrhea (OA) for PCOS.

Author	Year	Serum AMH Cut-Off (ng/mL)	Study Design	Area under the ROC Curve	Sensitivity (%)	Specificity (%)	AMH Assay
AMH alone in predicting PCOS							
Anand	2022	N/A	Meta-analysis	0.89	78	87	N/A
AMH replacing PCOM in Rotterdam Criteria for predicting PCOS							
Ahmed	2019	3.19	Case–control	0.927	78	100	AnshLabs
Eilertsen	2012	2.8	Case–control	0.992	94.6	97.1	Diagnostic System Laboratories
Lauritsen	2014	2.5	Cross-sectional	0.994	91.8	98.1	Immunotech Beckman Coulter
Sahmay	2014	3.8	Cross-sectional	0.97	100	88	Generation 2 Beckman Coulter
Saxena	2018	3.44	Cross-sectional	NA	86.67	100	Immunoconcept
AMH replacing OA in Rotterdam Criteria for predicting PCOS							
Ahmed	2019	3.19	Case–control	0.938	81	100	AnshLabs

## Data Availability

Not applicable.
